# Fungal Keratitis Due to *Fusarium lichenicola*: A Case Report and Global Review of *Fusarium lichenicola* Keratitis

**DOI:** 10.3390/jof7110889

**Published:** 2021-10-21

**Authors:** Isra Halim, Prabhakar Singh, Asim Sarfraz, Prathyusha Kokkayil, Binod Kumar Pati, Bhaskar Thakuria, Amit Raj

**Affiliations:** 1 Department of Microbiology, All India Institute of Medical Sciences (AIIMS), Patna 801507, India; dr.isra_micro@aiimspatna.org (I.H.); drprathyushak@aiimspatna.org (P.K.); drbinodkumarpati@aiimspatna.org (B.K.P.); drbhaskart@aiimspatna.org (B.T.); 2 Department of Ophthalmology, All India Institute of Medical Sciences (AIIMS), Patna 801507, India; prabhakar1aiims@gmail.com (P.S.); dramitraj@aiimspatna.org (A.R.)

**Keywords:** *Fusarium lichenicola*, *Cylindrocarpon lichenicola*, fungal keratitis, *Cylindrocarpon tonkinense*

## Abstract

*Fusarium* species are among the most commonly isolated causes of fungal keratitis. Most species of the genus *Fusarium* belong to *Fusarium solani* species complex (FSSC). *Fusarium lichenicola*, a member of the FSSC complex, is a well-established plant and human pathogen. However, reports of fungal keratitis due to Fusarium lichenicola have not been frequently reported. To the best of our knowledge, only twelve cases of *Fusarium lichenicola* keratitis have been reported in the past fifty years. Clinical cases of *Fusarium lichenicola* may have most likely been misidentified because of the lack of clinical and microbiological suspicion, as well as inadequate diagnostic facilities in many tropical countries where the burden of the disease may be the highest. We report a case of fungal keratitis caused by *Fusarium lichenicola* and present a global review of the literature of all cases of fungal keratitis caused by this potentially blinding fungus.

## 1. Introduction

Fungal keratitis (FK) is one of the leading causes of monocular blindness in the tropics [1]. India has witnessed an alarming rise in cases of FK in the past few decades. The percentage of FK as a subset of microbial keratitis rose from 51.9% in 1994 to a whopping 75.8% in 2012 in the country [2]. *Fusarium lichenicola*, a less commonly identified fungus, is a cause of relatively aggressive keratitis following ocular trauma. We describe a case of keratitis by *Fusarium lichenicola* and review the published literature on cases of FK caused by this fungus.

## 2. Case

**Figure 1:**
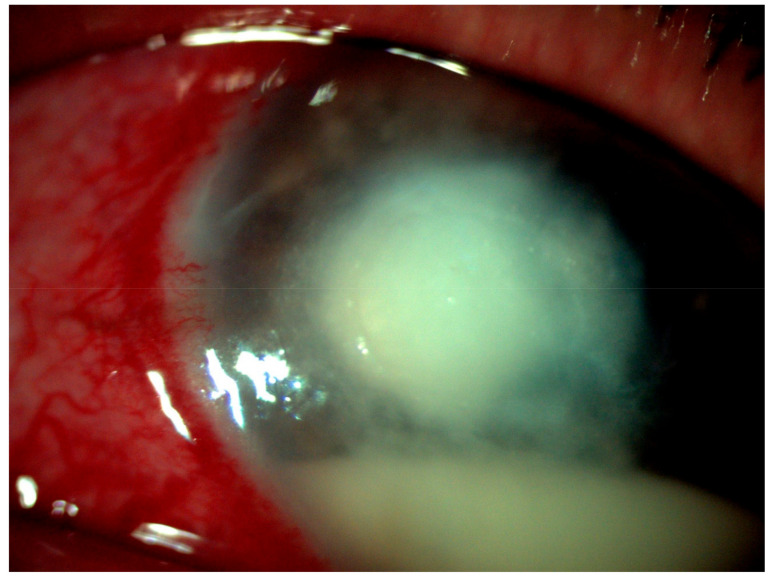
Greyish-white corneal infiltrate with feathery edges (5.5 mm × 6 mm) and multiple pin-head-sized peripheral satellite lesions with few tentacular extension.

A 28-year-old, immunocompetent male was presented to the ophthalmology outpatient department during the COVID-19 pandemic with complaints of severe pain, redness, and watering from the right eye for 20 days. The patient gave a history of foreign body in the right eye while driving his motorbike a month ago, following which he flushed his eyes with tap water. Given the nationwide lockdown, the patient did not immediately consult an ophthalmologist. Instead, he took topical over-the-counter medication, following which the redness and pain subsided. However, a week later, the pain and redness recurred for which he sought medical advice locally by a registered medical practitioner. The patient was started on two hourly medications, topical moxifloxacin (0.5%) and tobramycin (0.3%). Considering no improvement with the medications he was started on, the patient consulted a tertiary care academic hospital in north India. At presentation, the right eye visual acuity had been recorded to be hand movement, close to face. Right eyelid edema with mechanical ptosis was noted. On slit-lamp examination, the right eye showed a greyish-white infiltrate measuring 5.5 mm × 6 mm involving the temporal cornea. Multiple tiny pin-head-sized satellite lesions were observed all around the central dense infiltrate. Few linear infiltrates along with inferior endothelial exudates were also seen emanating from the central infiltrate (Figure 1). Circumciliary congestion with hypopyon measuring 2.5 mm was noted. Based on the presence of feathery edges of the infiltrates with surrounding satellite lesions, a clinical impression of fungal keratitis was made.

To establish the etiological diagnosis, the patient was subjected to right eye corneal scraping (for direct microscopy and culture) under strict aseptic conditions and immediately sent to the mycology section of the institutional laboratory. The direct microscopy in a 10% KOH mount showed hyaline, septate branching fungal hyphae. Based on microscopy findings, the patient was started on topical (hourly natamycin 5%) and systemic (ketoconazole 200 mg BD) antifungals for two weeks. Usually, fungal corneal ulcers respond well with topical and systemic antifungals if diagnosed early. However, an advanced disease threatening the limbus often requires therapeutic penetrating keratoplasty.

**Microbiology**

Corneal scraping was cultured on Sabouraud’s Dextrose Agar (SDA) slants (Hi-Media Laboratories Ltd., Mumbai, India) and incubated at 25 °C and 37 °C. The sample was also inoculated in C-streaks on 5% Sheep Blood Agar and Chocolate Agar. Within 48 h, similar fungal growth was obtained on all culture media. No bacterial growth was observed.

**Macroscopic features**

**Figure 2:**
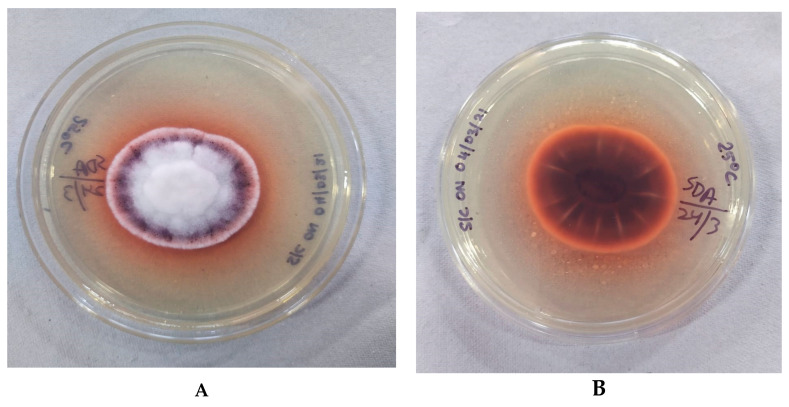
Macroscopic appearance of *Fusarium lichenicola* at 25 °C after 4 days of incubation. (**A**) Obverse showing white, short aerial hyphae with a pinkish-red rim; (**B**) reverse brown with a diffusible chestnut brown-red pigment.

Culture revealed velvety to floccose, white growth with a pinkish-brown rim on SDA. A diffusible chestnut red-brown pigment was also produced (Figure 2). At 25 °C, the fungus grew rapidly to a diameter of 40 mm by day 4 and up to 75 mm by day 7. The growth was comparatively slower on media incubated at 37 °C. 

**Microscopic Features**

**Figure 3:**
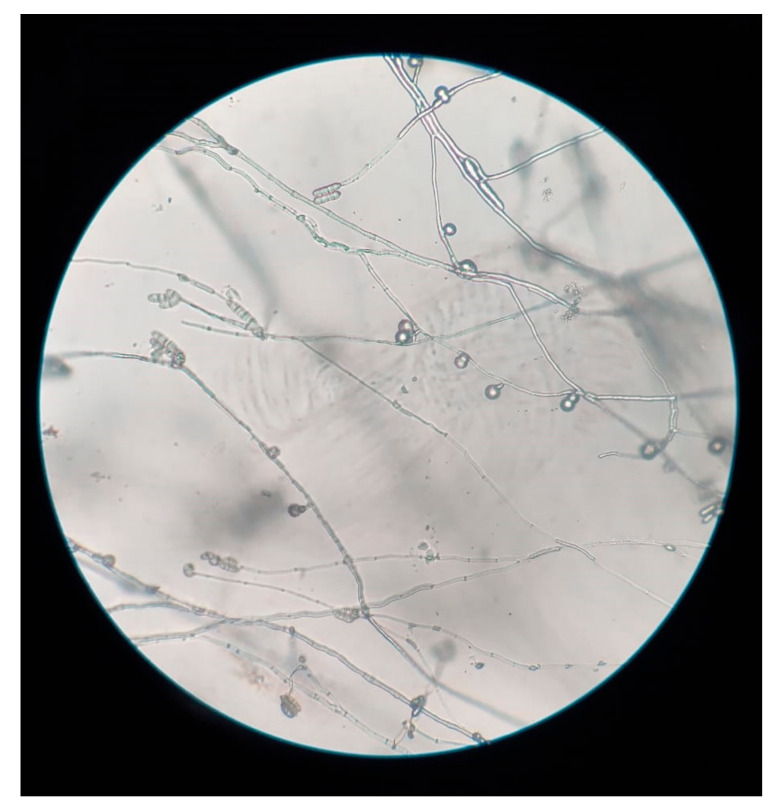
Slide culture of *Fusarium lichenicola*: Elongate conidiophores with terminal cylindrical macroconidia, and chlamydospores arising from short lateral branches (Magnification: 10×).

**Figure 4:**
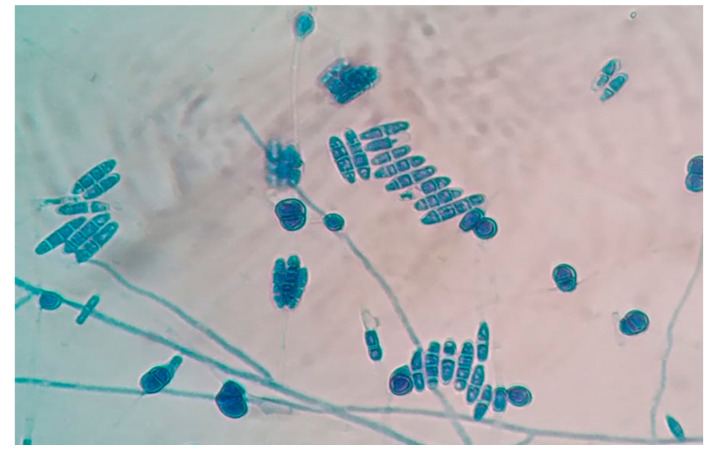
Lactophenol cotton blue mount of *Fusarium lichenicola* showing 2–4 celled macroconidia and multi-celled chlamydospores (Magnification: 40×).

A slide culture was set up from the growth and a lactophenol cotton blue mount (Hi-Media Laboratories Ltd., Mumbai, India) was prepared 48 h later. Microscopic examination revealed slender, septate fungal hyphae and numerous macroconidia (Figure 3). The macroconidia were ellipsoid to cylindrical, with 2–4 transverse septae and straight, smooth edges. The apex was blunt while the base was truncated with an offset pedicel. Macroconidia were arranged both singly and in clusters. No distinctive foot cells and no microconidia were appreciated. Numerous thick-walled, globose, chlamydospores originating from short lateral branches on the hyphae were also observed. While most of the chlamydospores were single-celled, 2–3 celled chlamydospores were also seen (Figure 4). 

**Sequence Analysis**

The fungus was identified to belong to the genus *Fusarium* based on macroscopic and microscopic features. However, the species was not conclusively established due to overlapping morphological features between *Fusarium lichenicola, Fusarium chlamydosporum,* and *Fusarium solani*. The identity was confirmed by sequencing the Internal Transcribed Spacer (ITS) regions, ITS 1, and ITS 4. The genetic material was analyzed using BigDye terminator cycle sequencing ready reaction kit, version 3.1 (Applied Biosystems, Foster City, CA, USA) on ABI 3730xl Genetic Analyzer (Applied Biosystems, Foster City, CA, USA). The sequence was used to carry out basic local alignment search tool (BLAST) analysis and compared with those in the NCBI GenBank. The isolate was finally identified as *F. lichenicola*, based on nucleotide homology of 99.64% with *Fusarium lichenicola* strain CNRMA10.15 isolate ISHAM-ITS_ID MITS1524 (Accession number: KP132217.1). The sequence obtained was submitted to the NCBI GenBank (Sequence number GenBank MZ413591).

The patient was followed up at our hospital for two weeks with no improvement on topical and systemic antifungals. He was subsequently referred to the regional corneal transplant center for therapeutic penetrating keratoplasty.

## 3. Discussion

FK is a potentially blinding condition with an estimated worldwide incidence of 1,051,787 (range: 736,251 to 1,367,323) cases per annum [1]. 

It causes perforation and entails surgical removal in up to a quarter of cases. FK causes mono-ocular blindness in nearly 60% of cases even with treatment [3,4,5]. A comprehensive review of FK showed that the highest proportion of cases reported are from India and Nepal [6]. *Fusarium* species and *Aspergillus* species are the two most common pathogens of FK from these countries. *Fusarium* keratitis alone constitutes nearly half of all cases of FK reported from India [7]. The common species of *Fusarium* identified in FK include *the F. solani species complex*, followed by the *F. dimerum species complex, F. fujikuroi species complex*, and *F. oxysporum species complex. Fusarium lichenicola*, as an etiology of FK, has been infrequently reported from the Indian subcontinent. To the best of our knowledge, this is the fourth reported case of *F. lichenicola* keratitis in India and the first from East India.

**Global Review of Literature:** ***F. lichenicola*** **keratomycoses from 1971 to May 2021**

A systematic search for cases of FK caused by *F. lichenicola* from 1971 to May 2021 was performed on the search engines Google Scholar, PubMed, and Scopus, using keywords ‘*Fusarium lichenicola*’, ‘*Cylindrocarpon lichenicola’*, ‘*Cylindrocarpon tonkinense*’, ‘fungal keratitis’, ‘mycotic keratitis’, and ‘keratomycoses’. Articles published in non-English languages were translated to English. The search revealed only 20 cases of all infections reported due to *F. lichenicola* in the world in the past 50 years. Of these, 11 were associated with FK. Other clinical presentations included cutaneous infections (*n* = 2) [8,9], mycetoma (*n* = 1) [10], intertrigo (*n* = 2) [11,12], peritonitis (*n* = 1) [13], onychomycosis (*n* = 1) [14], endophthalmitis (*n* = 1) [15] and disseminated infections (*n* = 2) [16,17]. 

Our review further describes *F. lichenicola* keratitis and its clinical and demographic features and emphasizes the importance of microbiological diagnosis of the disease. The clinico-epidemiological profile of patients diagnosed with *F. lichenicola keratomycoses* is tabulated below (Table 1).

*F. lichenicola* was first reported by Massalongo. The fungus belongs to the phylum ascomycetes, class sardariomycetes, order hypocreales and, family nectriaceae [28]. It belongs to the most common species complex of the genus, the *Fusarium solani* species complex (FSSC), where it is a member of subclade 16 of FSSC [29]. It was previously reported as *Cylindrocarpon lichenicola and* *Cylindrocarpon tonkinense*, but the original name *Fusarium lichenicola* C. B. Massalongo has been reestablished [30]. The fungus is a known soil and plant saprophyte in the tropical regions. Including this case, only 12 cases of *F. lichenicola* keratitis have been described in the past 50 years.

It is well established that the cases of mycotic keratitis are more common in regions closer to the equator [1]. Cases of *F. lichenicola* are also concentrated in the tropical countries (Table 1) close to the equator. However, four cases of *F. lichenicola* keratitis have been reported from temperate countries such as the United Kingdom, Germany, and France. Similar to other causes of fungal keratitis, *F. lichenicola* keratitis shows a male preponderance (8/12 = 67% cases). The mean age group of affliction is 52.4 years, ranging between 28 and 67 years. A study from South India reports a comparable age distribution in keratitis due to other filamentous fungi [7]. As seen among most other fungal keratitis cases, trauma with vegetative material often precede an episode of *F. lichenicola* keratitis, although in some cases, a history of trauma remained inconspicuous. 

**Challenges in clinical and microbiological diagnosis:**

Risk factors and clinical presentation of *F. lichenicola* keratitis were found to be no different from other causes of fungal keratitis. Our patient presented with feathery margins, satellite lesions and grey infiltrates. The clinical picture may be obscured by the use of over-the-counter self-medication, traditional eye medicine, and steroids before presentation to an ophthalmologist. No specific ophthalmic signs have been able to differentiate *F. lichenicola* keratitis from the presentation of keratitis due to other species of FSSC. Nevertheless, diagnostic algorithms such as those developed by Hoffman et al. may guide ophthalmologists in differentiating cases of *Fusarium* keratitis from other common causes of filamentous keratitides [2]. 

Despite overlapping clinical features of keratitis caused by members of the genus *Fusarium*, species identification is vital. This is because different species of *Fusarium* differ with regards to their antifungal susceptibility patterns and therapy may need to be tailored accordingly. Identifying less commonly reported fungal species will also help in estimating the true burden of the disease and help in the correct interpretation and development of current and future molecular diagnostic tests respectively.

Currently, fungal cultures remain the gold standard to diagnose keratitis caused by *F. lichenicola*. Microscopic findings, though specific, may lack sensitivity especially in the hands of inexperienced microbiologists. The authors postulate that *F. lichenicola* is perhaps misdiagnosed as *F. solani* or reported as *Fusarium* species in the absence of diagnostic tools for species confirmation. Our patient belonged to an economically backward region of the country with inadequate laboratory support. Molecular confirmation of the organism may not be feasible in such resource-limited settings. It is thus imperative for a microbiologist to be able to correctly identify *F. lichenicola* based on macroscopic and microscopic features alone. 

Unlike *F. solani*, *F. lichenicola* produces straight, rather than sickle-shaped or fusiform macroconidia. Conidiophores from which macroconidia arise are typically elongate in *F. lichenicola*, while they are comparatively short in *F. solani*. *F. lichenicola* also lacks microconidia and has pigmented chlamydoconidia. Their apical cells are rounded rather than tapering, while basal cells are truncated [9]. The macroconidia of *F. lichenicola* have lost their fusarial foot cell, whereas *F. solani* have minimally differentiated foot cells [29]. Despite the absence of foot cells, *F. lichenicola* are phylogentically classified within the FSSC. These characteristic morphological features should lead microbiologists towards the diagnosis of *Fusarium lichenicola.*

Our patient was started on oral antifungals, considering one dimension of the corneal ulcer was ≥5 mm, involving >50% of the corneal thickness. Ketoconazole 200 mg BD and topical natamycin 5% were given empirically. The Mycotic Ulcer Treatment Trials II advocate the addition of oral voriconazole to topical natamycin in cases of *Fusarium* keratitis [31]. The patient belonged to the lower socio-economic class and could not afford voriconazole. In a randomized controlled trial on effectiveness of oral ketoconazole vs. oral voriconazole as an adjunct to topical natamycin, both oral options were found to be equally good as far as duration and percentage of healed corneal ulcers was considered [32]. Successful treatment of *F. lichenicola* keratitis has been documented despite in vitro resistance against both Amphotericin B and Itraconazole [9,28]. In our case, antifungal susceptibility testing was not performed due to financial and resource constraints. Despite two weeks of antifungal treatment, the patient showed no improvement and was referred for therapeutic keratoplasty. The late presentation of the patient due to the nationwide lockdown could have contributed to the lack of response to conservative therapy. As for other causes of infective keratitis, the authors postulate that early diagnosis and management are essential to prevent long-term complications in cases of *F. lichenicola* keratitis.

*Fusarium lichenicola* has been reported in the soil and substrates in South India [33]. Similar epidemiological studies have not been undertaken to evaluate the presence of this saprophyte in other regions of the country. However, with favorable climatic conditions and a large farming population in the Northern belt of the country, more cases of *Fusarium lichenicola* may likely be diagnosed in the future. Accurate identification of the fungus up to the species level could help ophthalmologists look for characteristic clinical clues that may aid in early clinical suspicion of *F. lichenicola* keratitis. This review aims to generate awareness among ophthalmologists and microbiologists regarding the epidemiology, diagnosis, and management of *Fusarium lichenicola* keratitis. 

## Figures and Tables

**Table 1:** Global review of cases of Keratitis due to *Fusarium lichenicola*.

Case Number	Year Isolated	Place	Patient Age (in Years)	Gender	History of Trauma	Antifungal Therapy	Therapeutic Keratoplasty	Outcome	References
1	1971	Venezuela	46	Male	Muddy water	Topical Pimaricin 5%	Conjunctival flap	Improved	Laverde et al. [18]
2	1979	Japan	55	Female	Muddy water	Amphotericin B Clotrimazole 5 weeks both topical and systemic	No	Corneal perforation	Matsumoto et al. [19]
3	2001	Formosa, Argentina	30	Male	No	Topical natamycin 5%	No	Phthisis bulbi	Mangiaterra et al. [20]
4	2006	India	56	Male	Vegetative material	Topical natamycin 5%	Yes	Phthisis bulbi	Kaliamurthy J [21]
5	2006	Germany	61	Male	No (history of contact with Clematis plant)	Voriconazole 12 weeks both systemic and topical	No	Improved	Kaben et al. [22]
6	2006	Germany	71	Male	No	Topical Amphotericin B 6 weeks	No	Improved	Kaben et al. [22]
7	2009	United Kingdom	56	Female	No (keen Gardener, soft contact lens wearer)	Natamycin 5% drops every 2 h, oral voriconazole 200 mg twice daily	No	Complete resolution of ulcer with stromal scar	Mitra A et al. [23]
8	2017	Nigeria	67	Male	No (farmer)	Topical natamycin (5%), oral fluconazole (200 mg) daily	No	Corneal scar VA: no improvement (hand movements only)	Irek et al. [24]
9	2012	France	67	Female	Trauma with cloth fabric	Voriconazole, Amphotericin B	Yes	No recurrence after 1 year	Gaujoux T et al. [25]
10	1 case in a retrospective review (2005–2011)	Chandigarh, India	32	Male	Vegetative Material	Oral Itraconazole, natamycin 5%	No	Vascularized corneal opacity	Ghosh et al. [26]
11	2020	Kerala, India	60	Female	No	Oral ketoconazole, voriconazole, natamycin 5%	Yes	Stable with opaque graft requiring repeat TK	Shenoy et al. [27]
12	2021 *	Bihar, India	28	Male	Trauma with foreign body while driving a motorbike	Natamycin 5% topical, ketoconazole 200 mg systemic	Yes	Lost to follow up	Halim et al. *

* Current case.

## Data Availability

The data presented in this study are available in the article. The genome data of *Fusarium lichenicola* reported in this study will be provided on request by the corresponding author Asim Sarfraz.
